# Study on the Properties Changes of Reversible Invert Emulsion during the Process from O/W to W/O with Alkali

**DOI:** 10.3390/molecules29010062

**Published:** 2023-12-21

**Authors:** Fei Liu, Yongfei Li, Xiaqing Li, Xuewu Wang

**Affiliations:** 1 College of Petroleum Engineering, Shandong Institute of Petroleum and Chemical Technology, Dongying 257061, China; 2016001@sdipct.edu.cn; 2 Chemistry and Chemical Engineering, Xi’an Shiyou University, Xi’an 710065, China; yfli@xsyu.edu.cn; 3 Petroleum Engineering Technology Research Institute of Shengli Oilfield Company, SINOPEC, Dongying 257000, China; lixiaqing.slyt@sinopec.com

**Keywords:** pH-responsive, phase inversion, microstructure, reversible invert emulsion

## Abstract

The reversible emulsion drilling fluid system combines the advantages of both oil-based and water-based drilling fluids, which can achieve ideal results in different stages of drilling and completion, and the system can be reused to effectively reduce costs. However, the research on reversible emulsions mainly focuses on the development of new reversible emulsifiers, while the specific phase transformation mechanism of reversible emulsion systems is still unclear. In this paper, a stable reversible emulsion was prepared using the reversible emulsifier DMOB as a raw material, and the reversible emulsion performance of the alkali response from the O/W emulsion phase to the W/O emulsion was studied. The microstructure of reversible emulsions was studied by a microscope, a cryogenic transmission electron microscopy, and a laser particle size analyzer. The changes in macroscopic properties of reversible emulsions in the process of alkali conversion were studied by pH, conductivity, demulsification voltage, static stability, viscosity, rheology, and other indicators, and the conversion mechanism of reversible emulsions from O/W emulsion ⟶ bicontinuous structure ⟶ O/W/O emulsion ⟶ W/O emulsion was clarified. The details are as follows: in the first stage, when the amount of NaOH ≤ 0.43 vol.%, the overall particle size of the emulsion decreases first and then increases with the increase in NaOH dosage. In the second stage, when the amount of NaOH was 0.45 vol.%, a double continuous structure was formed inside the emulsion. In the third stage, when the amount of NaOH is 0.48 vol.%, the O/W/O emulsion is formed, and with the increase in stirring time, the internal oil droplets gradually accumulate and are discharged from the water droplets, and finally, the W/O emulsion is formed. In the fourth stage, when the dosage of 0.50 vol.% ≤ NaOH ≤ 5.00 vol.%, the W/O emulsion was formed, and with the increase of NaOH dosage, the structure and compactness between water droplets increased first and then decreased. In the whole process, with the increase in the amount of NaOH solution, the total particle size of the emulsion first decreased and then increased.

## 1. Introduction

Emulsions can be divided into oil-in-water (W/O) emulsions, water-in-oil emulsions (O/W), and multiple emulsions [1,2,3]. In a sense, it is a combination of W/O emulsion and O/W emulsion [4]. Reversible emulsions can achieve reversible conversion between W/O emulsions and O/W emulsions by controlling influencing factors, thereby harnessing the advantages of different types of emulsions. Currently, the main factors affecting reversible emulsions include pH [5,6,7], temperature, salinity [8], pH-temperature synergy [9], and light. Among them, pH-sensitive reversible emulsions have attracted widespread attention in the drilling fluid field [10,11,12,13,14].

**Figure 1:**
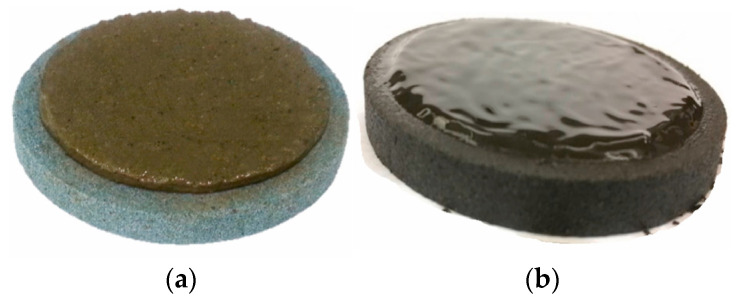
Filter cake formed with water-based drilling fluids and oil-based drilling fluids [19]. (**a**) water-based drilling fluids; (**b**) oil-based drilling fluids.

Drilling fluids are primarily divided into oil-alkaline and water-alkaline. The oil-alkaline can withstand high temperatures [15], exhibit good lubrication, possess strong inhibitory properties [16], and have the ability to resist salt and calcium contamination. They can effectively reduce damage to oil and gas reservoirs. Therefore, during the drilling phase, oil-alkaline drilling fluids are superior to water-alkaline [17]. The oil-alkaline drilling fluid has become an important tool for drilling different wells, including the deep/ultra-deep, the extended reach, horizontal, and various complex formations [18]. However, they also have some drawbacks. During the completion phase, they can cause difficulties in filter cake removal (Figure 1) [19], weak bonding strength between cement and formation, and challenges in handling oil-laden cuttings [20,21,22]. Water-alkaline drilling fluids are evidently superior to oil-alkaline during the completion phase [23,24,25].

**Figure 2:**
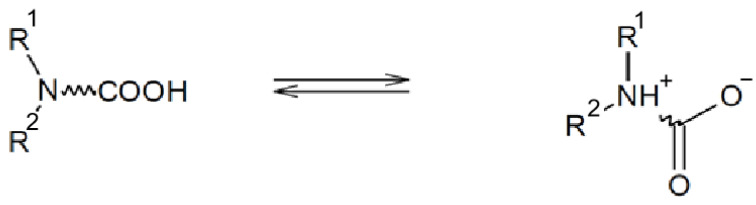
General representation of the surfactant [26].

Reversible emulsified drilling fluid systems can achieve the conversion between W/O and O/W emulsions by controlling the system’s acidity and alkalinity. This means that during the drilling and completion stages, the drilling fluid can exhibit the properties of oil-alkaline drilling fluids. However, by adding water-soluble acid during the completion phase and subsequent operations, it can transform into an O/W emulsified drilling fluid. This allows for the combination of the advantages of oil-alkaline and water-alkaline drilling fluids to achieve the desired drilling and completion effects (Figure 2) [26,27].

**Figure 3:**
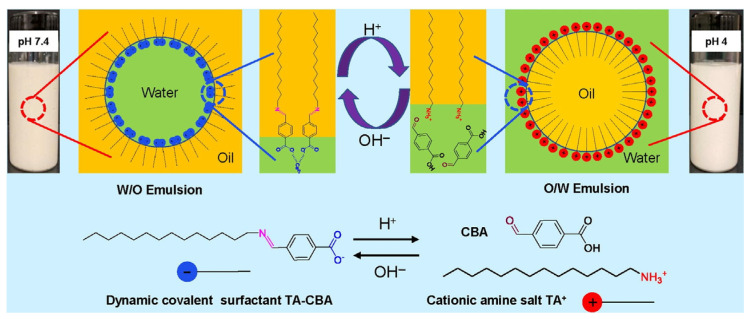
The pH-responsive mechanism of the TA-CBA-stabilized emulsions [28].(The green area is water, the yellow area is oil).

Currently, research on reversible emulsified drilling fluids mainly focuses on the development of reversible emulsifiers and the construction of reversible emulsified drilling fluid systems. For example, Wang et al. synthesize a novel dynamic covalent surfactant (TA-CBA) that can precisely manipulate the emulsion type by responding to acid/alkali stimuli through the Schiff alkali reaction between 1-tetradecylamine (TA) and 4-carboxybenzaldehyde (CBA) (Figure 3) [28].

Li et al. prepare a pH-responsive drilling fluid with a temperature resistance of up to 150 °C resistance and a density of 1.5 g/cm^3^ using mixed emulsifiers [29]. Ren et al. prepared a reversible invert emulsion drilling fluid to achieve good rheological and filtration properties at a low oil-water ratio of 50:50–60:40, a high density of 1.8–2.1 g/cm^3^, and a high temperature of 180–200 °C [30]. However, the mechanism of phase inversion for reversible invert emulsion drilling fluids is not yet thoroughly understood. 

Although reversible emulsified drilling fluids can harness the advantages of both water-alkaline and oil-alkaline, their usage cost is relatively high. The alkali phase inversion (from W/O to O/W) property can enable their repeated use. This property is crucial for reducing the cost and promoting their wider application. Considering that the basis of reversible emulsion drilling fluid is a reversible emulsion and the reversible phase mechanism has not been deeply understood, this study focuses on the investigation of the alkali phase inversion mechanism in reversible emulsions, which has not yet been publicly reported. The change in demulsification voltage and conductivity of reversible emulsions with the increase of alkali dosage was studied to determine the phase transition point of reversible emulsions under different conditions. The stability of a water-in-oil emulsion was characterized by a breaking voltage. The water phase continuity of oil-in-water emulsions was characterized by the conductivity of W/O emulsions. By studying the static stability of reversible emulsion obtained by changing the amount of alkali, the stability of reversible emulsion formed at each stage in the process of alkali conversion was characterized, and the changes of reversible emulsion at each stage in the process of reversible phase were analyzed. Since the change in the rheology of emulsions is closely related to the change in microstructure [31,32,33,34,35], the rheological properties of emulsions formed at each stage of the transition process were evaluated, verified with the microstructure changes of the emulsions observed by optical and electron microscopy, and identified as the related mechanism of the phase transformation of reversible emulsions with alkali (Table 1).

## 2. Results and Discussion

**Figure 4:**
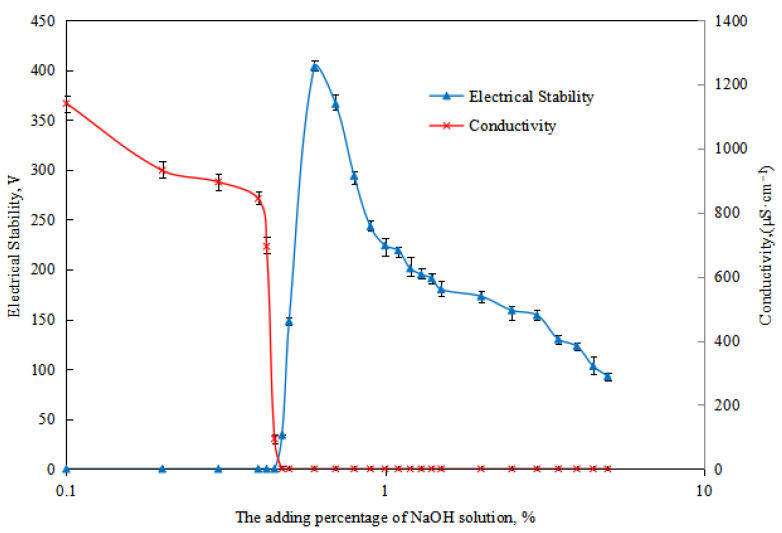
The effect of the NaOH solution on the conductivity and breaking voltage of the emulsion.

The basic properties of the initial O/W emulsion (Type II emulsion) were tested. The parameters of W/O emulsion with NaOH solution were as follows: breaking voltage (0 V), electrical conductivity (1002 µS cm^−1^), oil separation rate after 24 h of settling (0.00 vol.%), water separation rate after 24 h of settling (0.00 vol.%), and pH value of 6.0. By studying the variation of emulsion breaking voltage and conductivity with the amount of NaOH solution (Figure 4) and combining it with the observation of the emulsion’s morphology, the phase transition point of the reversible emulsion with alkali addition was analyzed. It was determined that when the amount of NaOH solution reached 0.43 vol.%, the emulsion remained as an O/W emulsion. When the amount of NaOH solution is between 0.45 vol.% and 0.48 vol.%, the emulsion is in a transitional phase. When the amount of NaOH solution reached 0.50 vol.%, the emulsion transitioned into a W/O emulsion. 

The fundamental reason for the phase transition of the reversible emulsion upon alkali addition is the influence of the alkali solution on the pH value of the emulsion system, which in turn affects the properties of the reversible emulsifier, ultimately leading to the alkali-induced phase transition of the reversible emulsion. When the amount of NaOH solution reached 0.50 vol.%, the reversible emulsion underwent a phase transition. To study the phase transition process of the emulsion at 0.50 vol.% NaOH solution, it was found that the phase transition occurred too rapidly to be studied by adjusting the stirring time. However, there was a clear transitional stage observed in the emulsion during the process of changing the amount of NaOH solution. Therefore, this transitional stage can be used to investigate the phase transition process of the reversible emulsion by varying the amount of NaOH solution.

**Figure 5:**
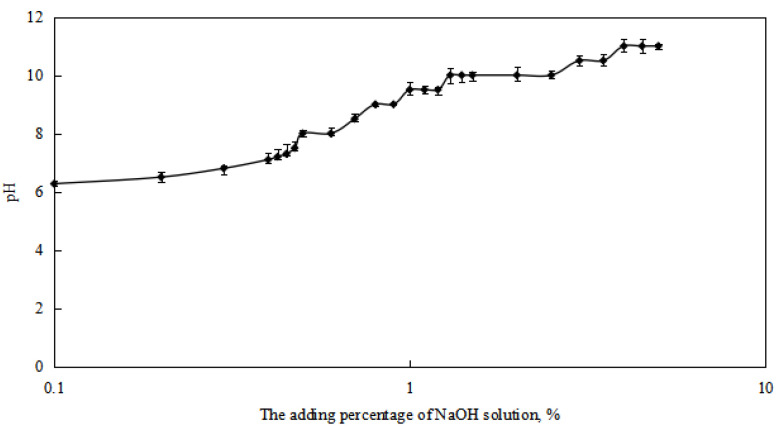
The effect of the NaOH solution on the pH value of the reversible emulsion.

The variation of the pH value of the reversible emulsion system with increasing amounts of NaOH solution was studied (Figure 5). It was found that when the amount of NaOH solution reached 0.50%, corresponding to a pH value of 8.0 in the emulsion system, the emulsion underwent an alkali-induced phase transition. When the amount of NaOH solution reached 0.48%, corresponding to a pH value of 7.5 in the emulsion, the emulsion was in a transitional stage. Therefore, the pH value of 8.0 was determined as the pH value at which the reversible emulsion undergoes a complete alkali-induced phase transition.

Based on the preliminary experimental results of the alkali-induced phase transition in the reversible emulsion, the process can be divided into three stages: Stage 1: O/W emulsion phase. In this stage, the variation of the properties of the reversible emulsion with increasing amounts of NaOH solution is studied when the amount is less than 0.43 vol.%. Stage 2: Transitional stage. In this stage, the amount of NaOH solution ranges between 0.45 vol.% and 0.48 vol.%. The variation of the emulsion properties with increasing amounts of NaOH solution is studied during this transitional stage. Stage 3: W/O emulsion phase. In this stage, the variation of the properties of the reversible emulsion with increasing amounts of NaOH solution is studied when the amount is greater than 0.50 vol.%.

### 2.1. O/W Emulsion Phase

In the O/W emulsion phase of the alkali-induced phase transition of the reversible emulsion, under the condition of a stirring time of 5 min and varying the amount of NaOH solution (NaOH solution amount < 0.43 vol.%), the change in viscosity of the emulsion system was studied.

**Figure 6:**
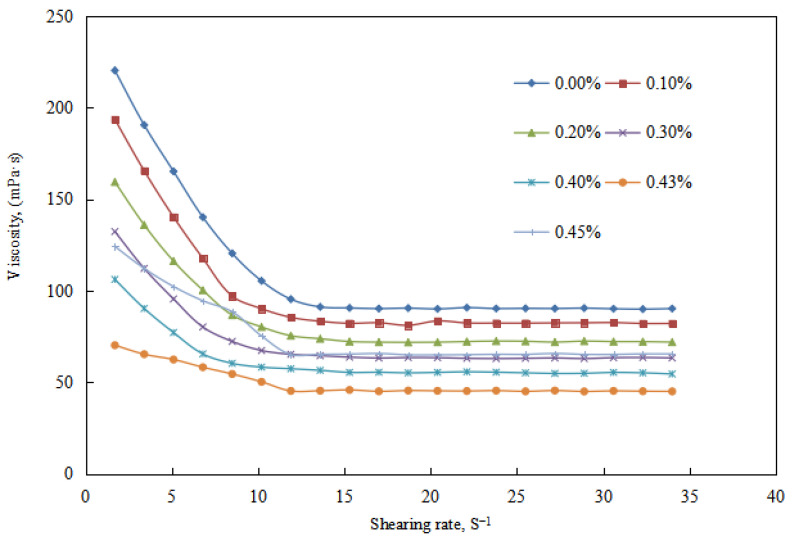
The effect of the NaOH solution on the viscosity of the O/W emulsion.

**Figure 7:**
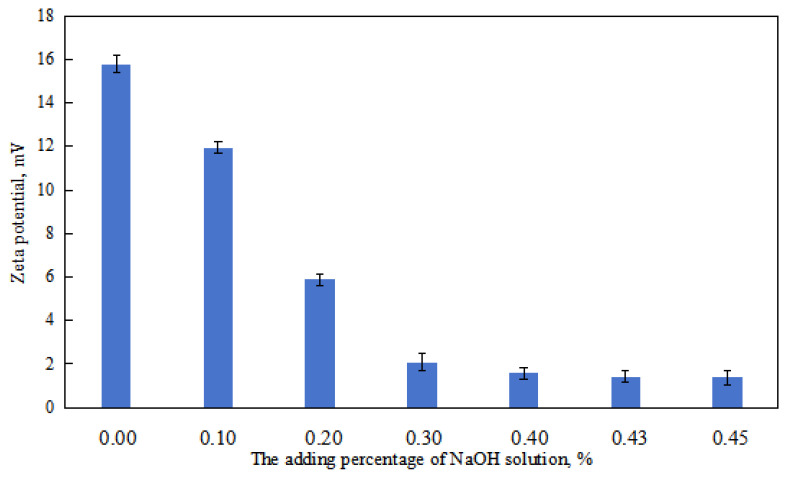
The effect of alkali on the Zeta potential of the W/O (O/W) emulsion.

**Figure 8:**
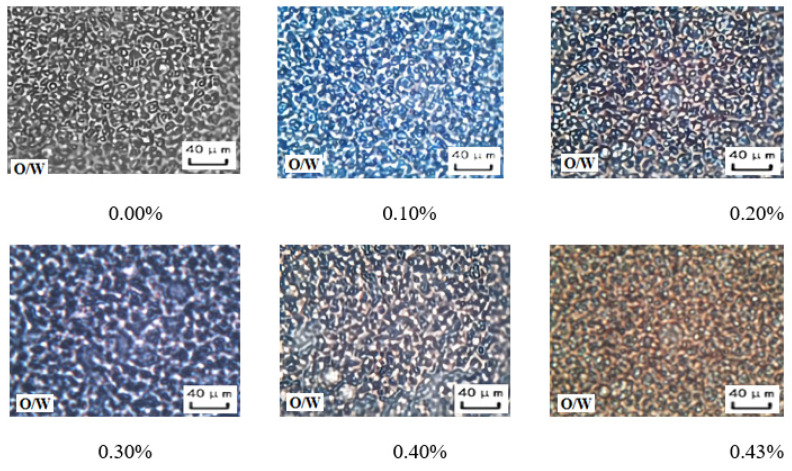
The effect of the NaOH solution on the micro-morphology of the O/W emulsion.

**Figure 9:**
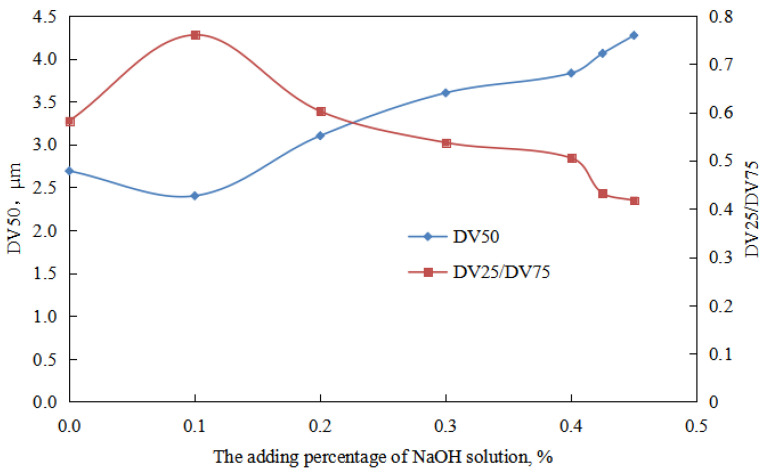
The effect of the NaOH solution on the droplet size characteristics of a W/O emulsion.

① When the amount of NaOH solution is 0–0.10 vol.%, the viscosity of the O/W emulsion system decreased with the increase NaOH in dosage (Figure 6). This can be attributed to the decrease in the charge of the oil droplets in the O/W emulsion with an increase in the amount of NaOH solution (Figure 7). Additionally, the overall droplet size of the O/W emulsion slightly decreased with the increase amount of NaOH solution, and the uniformity of the droplet size distribution in the emulsion increased (Figure 8 and Figure 9). Taking these factors into consideration, the viscosity of the O/W emulsion system decreases as the amount of NaOH solution increases.

When the amount of NaOH solution is 0.10–0.43 vol.%, the viscosity of the O/W emulsion system decreased with an increase in the amount of NaOH solution (Figure 7). This can be attributed to the decrease in the charge of the oil droplets in the O/W emulsion with an increase in the amount of NaOH solution (Figure 6). Additionally, with an increase in the amount of NaOH solution, the overall droplet size of the O/W emulsion gradually increased, and the uniformity of the droplet size distribution in the emulsion decreased (Figure 8 and Figure 9). The impact of changes in the charge of the emulsion droplets and droplet size on the emulsion viscosity outweighed the impact of the uniformity of droplet size distribution on the emulsion viscosity. Therefore, the viscosity of the emulsion decreased with an increase in the amount of NaOH solution.

The microstructure of the aqueous oil-in-water emulsion was analyzed with the increase in the amount of sodium hydroxide solution in the reversible emulsion phase conversion process (Figure 8). It was found that in the stage where the amount of NaOH solution was less than 0.43 vol.%, there was no specific structure between the droplets of the O/W emulsion, and the overall droplet size of the emulsion showed a trend of first decreasing and then increasing. When the amount of NaOH solution is 0.10 vol.%, the overall particle size of emulsion droplets is the smallest (Figure 9).

**Figure 10:**
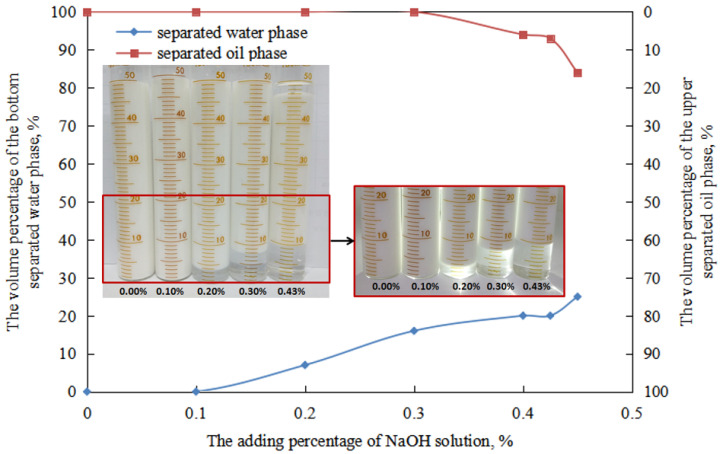
The effect of the NaOH solution on the standing stability of the O/W emulsion.

For emulsions prepared under different NaOH solution dosages (W/O emulsion), after standing for 24 h, the change of oil separation rate with the increase in NaOH solution dosages can be divided into two stages (Figure 10).

① When the amount of sodium hydroxide solution is low (≤0.30 vol.%), the oil separation rate is 0 vol.%. This is because the overall droplet size of the O/W emulsion is small, and it is distributed relatively uniformly in the aqueous phase. The emulsion has a larger interfacial area and higher total energy. The resistance to droplet coalescence is significant, and the droplets in the emulsion have a high charge (Figure 6), which further increases the resistance to droplet coalescence. As a result, the O/W emulsion droplets remain in a relatively stable state, leading to a zero oil separation rate.

② When the amount of sodium hydroxide solution is high (0.40–0.43 vol.%), the oil separation rate of the O/W emulsion increases with an increase in the amount of NaOH solution. This is because the stabilizing effect of the emulsifier on the O/W emulsion becomes poor. Additionally, the charge of the emulsion droplets is lower, and the overall morphology and uniformity of droplet size distribution in the O/W emulsion deteriorate with an increase in the amount of NaOH solution. The charge of the emulsion droplets decreases with an increase in the amount of NaOH solution. In other words, the stability of the O/W emulsion droplets is poor during this stage, and their stability worsens with an increase in the amount of NaOH solution. Hence, the oil separation rate increases within this range with an increase in the amount of NaOH solution.

For emulsions prepared under different NaOH solution dosages (O/W emulsion), after standing for 24 h, the change of water separation rate with the increase in NaOH solution dosages can be divided into two stages (Figure 10).

① When the amount of sodium hydroxide solution is low (≤0.30 vol.%), the water separation rate of the O/W emulsion remains at 0.00 vol.%. This is because the overall droplet size of the O/W emulsion is small, and the emulsion droplets have a higher charge. The O/W emulsion droplets remain in a relatively stable state, resulting in a zero water separation rate.

② When the amount of sodium hydroxide solution is high (0.20–0.43 vol.%), the water separation rate of the O/W emulsion increases with an increase in the amount of NaOH solution. This is because the overall morphology and uniformity of droplet size distribution in the O/W emulsion deteriorate. Additionally, the charge of the emulsion droplets decreases, indicating a decrease in the stability of the O/W emulsion. This leads to a greater tendency for the emulsion droplets to aggregate and pack tightly. Moreover, within the higher NaOH solution range (0.40–0.43 vol.%), the oil separation rate of the O/W emulsion increases with an increase in the amounts of NaOH solution, further contributing to an increase in the water separation rate.

### 2.2. Transition Stage

The variation of viscosity with the amount of NaOH solution (0.45 and 0.48 vol.%) was studied in the transitional stage of the emulsion system.

**Figure 11:**
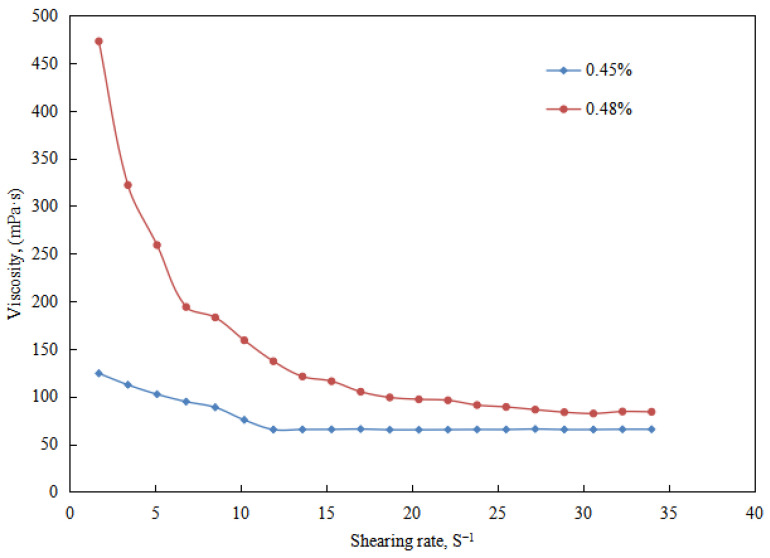
The effect of the NaOH solution on the viscosity of the emulsion in a transitory stage.

**Figure 12:**
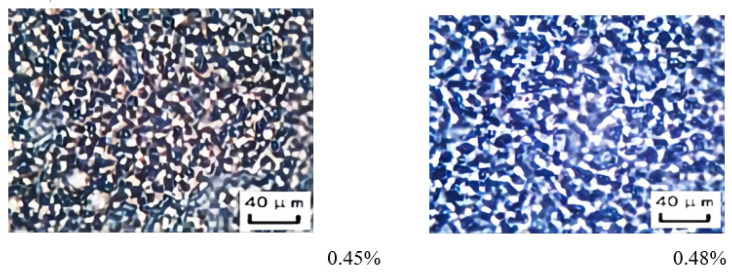
The effect of NaOH solution on the microstructure of the emulsion in a transitory stage.

The results indicate (Figure 11) that the emulsion system formed with a 0.48 vol.% NaOH solution exhibits higher viscosity compared to the emulsion system formed with 0.45 vol.%. The experimental groups with a NaOH solution dosage between 0.45 vol.% and 0.48 vol.% were analyzed, all of which were in the transition stage of partial emulsion demulsification, and specific tests were conducted for emulsion groups with a sodium hydroxide solution dosage of 0.45 vol.%. The breakdown voltage of the emulsion is 0 V, and the conductivity is 94 µS cm^−1^. For the emulsion group with a NaOH solution amount of 0.48 vol.%, the emulsion breakdown voltage is 34 V, and the conductivity is 0 µS cm^−1^, indicating that although partial emulsion breakdown occurs, the main composition is a W/O emulsion and an O/W-in-water emulsion with some structured water droplets (Figure 12). Therefore, the emulsion system formed with a NaOH solution amount of 0.48 vol.% exhibits higher viscosity than that formed with 0.45 vol.%, which is consistent with the experimental results (Figure 11).

**Figure 13:**
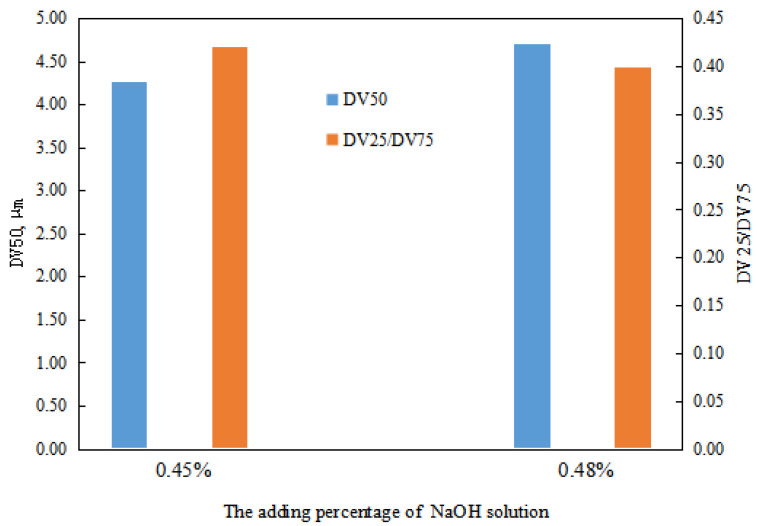
The effect of the NaOH solution on the emulsion-particle size value in a transitory stage.

The particle size of the transition phase system of the emulsion was analyzed when the dosage of NaOH solution was between 0.45 and 0.48 vol.%. The experimental results revealed that the overall droplet size of the emulsion system formed with a NaOH solution amount of 0.48 vol.% is larger than that of the emulsion system formed with 0.45 vol.% (Figure 13). Furthermore, the droplet size distribution of the emulsion system formed with a NaOH solution amount of 0.45 vol.% is comparatively more concentrated than that of the emulsion system formed with 0.48 vol.% (Figure 13). The main reason for this is the presence of structured water droplets between the W/O emulsion. Although the emulsion was diluted before conducting the particle size distribution analysis, some of the inter-droplet structures remained intact. As a result, the emulsion system formed with a NaOH solution amount of 0.48 vol.% exhibits larger droplet sizes and a more dispersed droplet size distribution compared to that formed with a NaOH solution amount of 0.45 vol.%.

**Figure 14:**
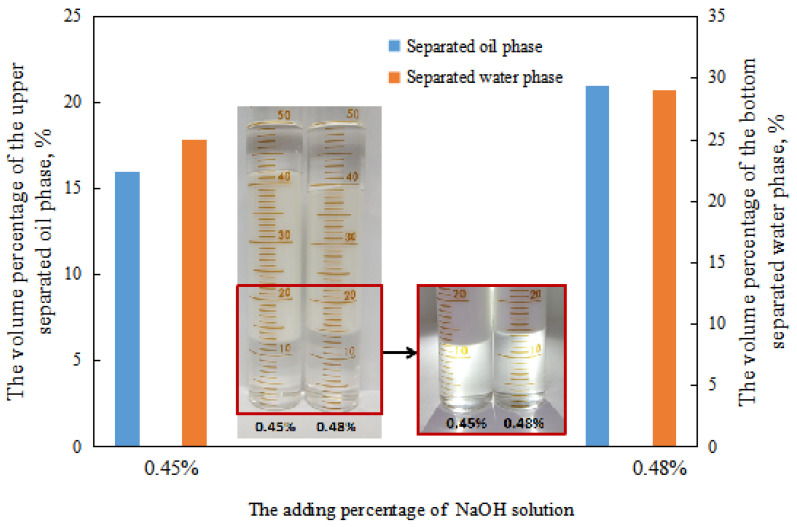
The effect of the NaOH solution on the standing stability of the emulsion in a transitory stage.

In the process of alkali conversion, the emulsion formed in the transition stage was partially demulsified when the dosage of sodium hydroxide solution was between 0.45 and 0.48 vol.% after standing for 24 h. Consequently, a significant separation of the water phase and oil phase occurs during the settling process, which is consistent with the experimental results (Figure 12 and Figure 14).

**Figure 15:**
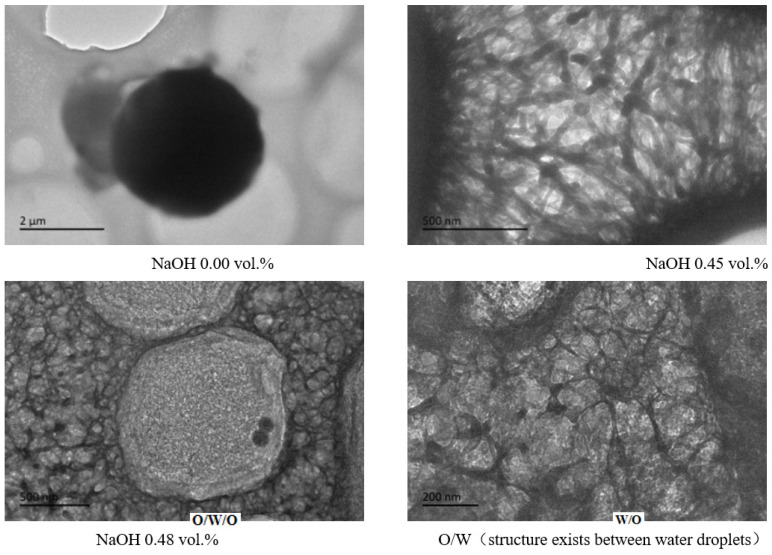
Schematic diagram of the microscopic transformation process of reversible emulsion with alkali.

**Figure 16:**
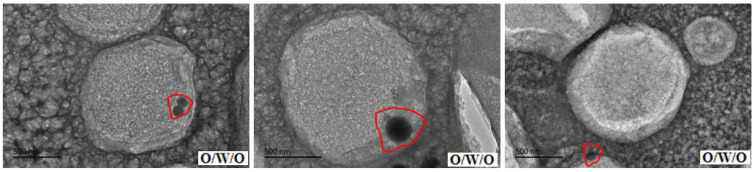
The change in the microstructure of the O/W/O emulsion during the transitory stage.(The area marked by the red line represents the change process of oil droplet in O/W/O emulsions).

Through further study of the emulsion system in the transition phase of reversible emulsion adding alkali to phase, we have a deeper understanding of the microscopic process of reversible emulsion adding alkali to phase conversion (Figure 15). When the amount of NaOH solution was 0.45 vol.%, the oil droplets in the W/O emulsion had a bicontinuous structure. When the amount of NaOH solution was 0.48 vol.%, a W/O emulsion was formed, and the innermost phase oil droplets were coalescing and gradually discharged from the water droplets. When the innermost phase oil droplets completed the coalescing and expulsion processes, the W/O emulsion was formed. The water droplets in the water-in-oil emulsion will gradually be separated from large water droplets to small water droplets with the increase in the amount of acid, forming a stable water-in-oil emulsion. In order to have a deeper understanding of the process of phase transformation of reversible emulsions by adding alkali, the differences between O/W/O emulsions prepared under different stirring times were comprehensively observed and analyzed. It was found that the number of oil droplets in the innermost phase of the oil-in-water emulsion decreased and the volume increased with the increase in stirring time. Therefore, in the process of transforming O/W/O emulsion into W/O emulsion, the innermost oil droplets polymerized before the innermost oil droplets were discharged from the water droplets (Figure 16).

### 2.3. W/O Emulsion Stage

The variation of viscosity in the emulsion system during the W/O emulsion stage (0.50–5.00 vol.%) of the alkali-induced phase inversion process in the reversible emulsion was studied.

**Figure 17:**
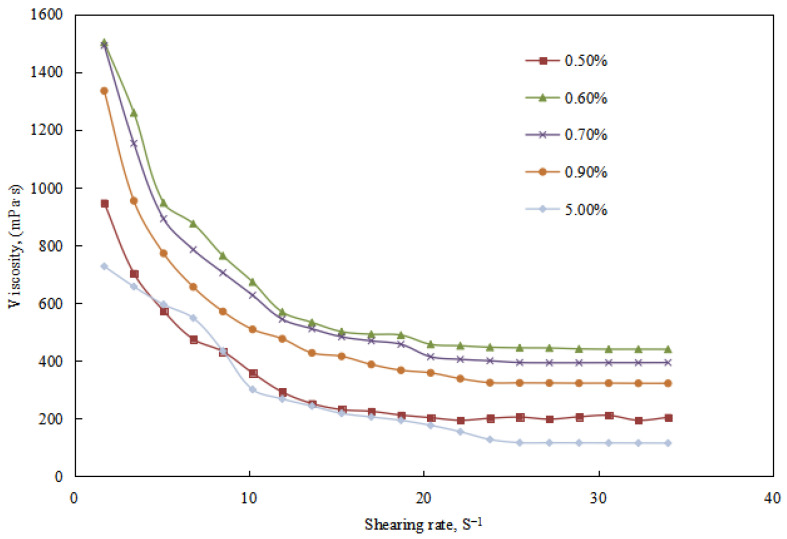
The effect of the NaOH solution on the viscosity of the W/O emulsion.

**Figure 18:**
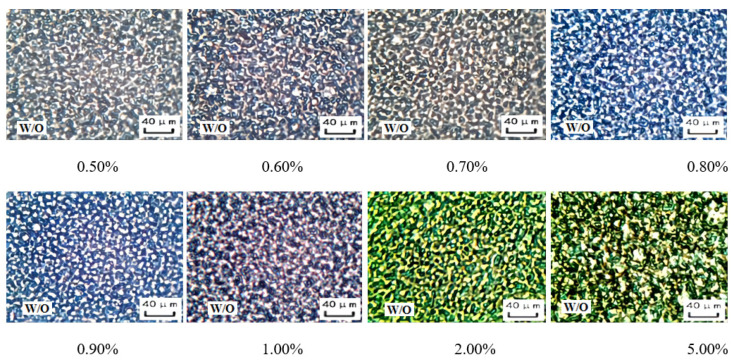
The effect of the NaOH solution on the micro-morphology of the W/O emulsion.

**Figure 19:**
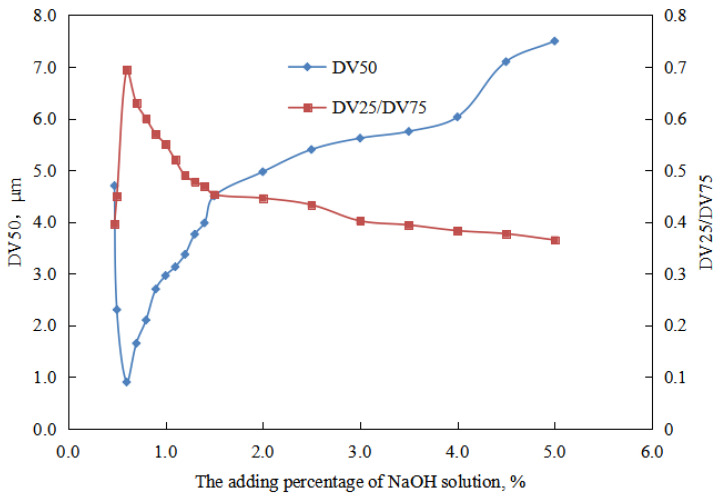
The effect of the NaOH solution on the W/O emulsion-particle size characteristic value.

In this stage (0.50 vol.% ≤ amount of NaOH solution ≤ 5.00 vol.%), the viscosity of the emulsion system initially increased and then decreased with the increase of the amount of NaOH solution (Figure 17). The W/O emulsion exhibits the highest viscosity at a NaOH solution amount of 0.60 vol.%. From the perspective of the emulsion’s microscopic structure and droplet size distribution (Figure 18 and Figure 19), it can be explained that the compactness of the water droplet structure between the emulsion droplets initially increases and then decreases with an increasing amount of NaOH solution. The tightest water droplet structure is observed at a NaOH solution amount of 0.60 vol.%. Additionally, the overall droplet size of the emulsion initially decreases and then increases with an increasing amount of NaOH solution, reaching the minimum at a NaOH solution amount of 0.60 vol.%. The uniformity of the droplet distribution initially increases and then decreases with an increasing amount of NaOH solution, with the most uniform droplet distribution observed at a NaOH solution amount of 0.60 vol.%.

Combining these experimental results with the viscosity test results of the reversible emulsion, it can be concluded that in this stage (0.50 vol.% ≤ amount of NaOH solution ≤ 5.00 vol.%), the effect of the compactness of the water droplet structure and the overall droplet size on the emulsion viscosity is more significant than the effect of the uniformity of the droplet distribution. Comparing the trend of the emulsion breaking voltage in the reversible emulsion, it is observed that the emulsion breaking voltage initially increases and then decreases with an increasing amount of NaOH solution. The maximum value is reached at a NaOH solution amount of 0.20 vol.%, indicating that the emulsion is most stable at this point. This observation is consistent with the results obtained from the microscopic structure analysis and droplet size distribution test of the reversible emulsion.

**Figure 20:**
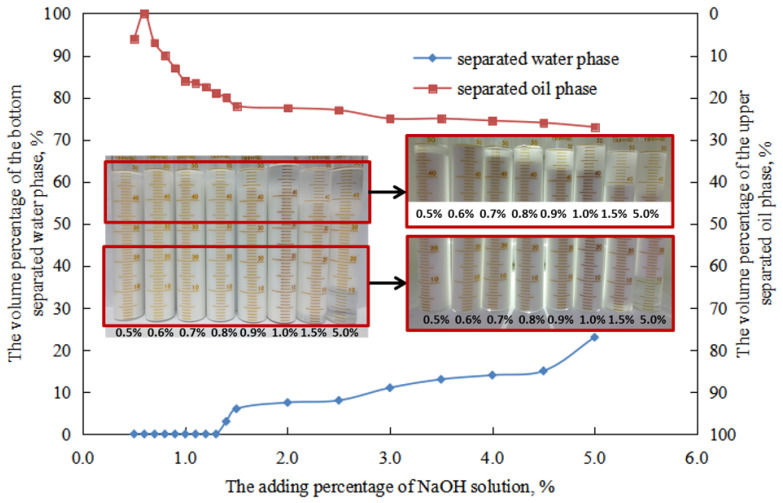
The effect of the NaOH solution on the standing stability of the W/O emulsion.

The variation of water separation rate after 24 h of sedimentation in the O/W emulsion prepared under different solution conditions can be divided into two stages (Figure 20).

When the amount of NaOH solution is low (0.5–1.0 vol.%), the water separation rate in the W/O emulsion is 0.0 vol.%. This is because the water droplets in the W/O emulsion maintain a relatively tight structure, indicating better stability. Therefore, there is no droplet destruction or water release in the W/O emulsion within this range.

When the amount of NaOH solution is high (2.0–5.0 vol.%), the water separation rate in the W/O emulsion increases with an increase in the amount of NaOH solution. This is because as the amount of NaOH solution increases, the compactness of the water droplet structure in the W/O emulsion decreases, resulting in decreased stability. Consequently, after 24 h of sedimentation, the water separation rate in the W/O emulsion increases with an increase in the amount of NaOH solution.

The variation of oil separation rate after 24 h of sedimentation in the W/O emulsion prepared under different NaOH solution conditions can be divided into three stages (Figure 20):

When the amount of NaOH solution is low (0.5–0.6 vol.%), the oil separation rate in the W/O emulsion decreases with an increase in the amount of NaOH solution. This is because, as the amount of NaOH solution increases, the compactness of the water droplet structure in the W/O emulsion increases. The difficulty of achieving tight stacking through the destruction of the water droplet structure between the oil droplets also increases, leading to a decrease in the oil release rate. Additionally, the stability of the water droplets in the W/O emulsion increases, resulting in fewer droplets being destroyed and released in the aqueous phase. The increase in the volume of the aqueous phase within the W/O emulsion also contributes to a decrease in the oil release rate with an increase in the amount of NaOH solution.

When the amount of NaOH solution is 0.60 vol.%, the oil separation rate in the W/O emulsion is 0. This is because the water droplet structure in the W/O emulsion can maintain a relatively tight state during this stage, indicating good stability. The structure of the water droplets in the W/O emulsion will not be destroyed during the 24 h sedimentation period.

When the amount of NaOH solution is between 0.7 and 5.0 vol.%, the oil separation rate in the W/O emulsion increases with an increase in the amount of NaOH solution. This is mainly due to the decreased compactness of the water droplet structure in the W/O emulsion. The difficulty of achieving tight stacking through the destruction of the water droplet structure decreases, leading to an increase in the oil release rate. Additionally, with an increase in the amount of NaOH solution, more water droplets are destroyed and released as the aqueous phase during this process. The decrease in the volume of the aqueous phase within the W/O emulsion also contributes to an increase in the amount of oil separation.

**Figure 21:**
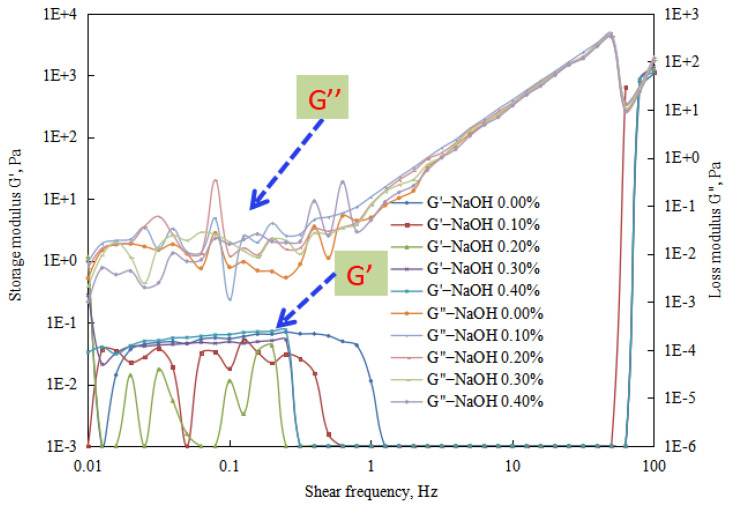
The effect of the NaOH solution on the rheological property of the O/W emulsion.

**Figure 22:**
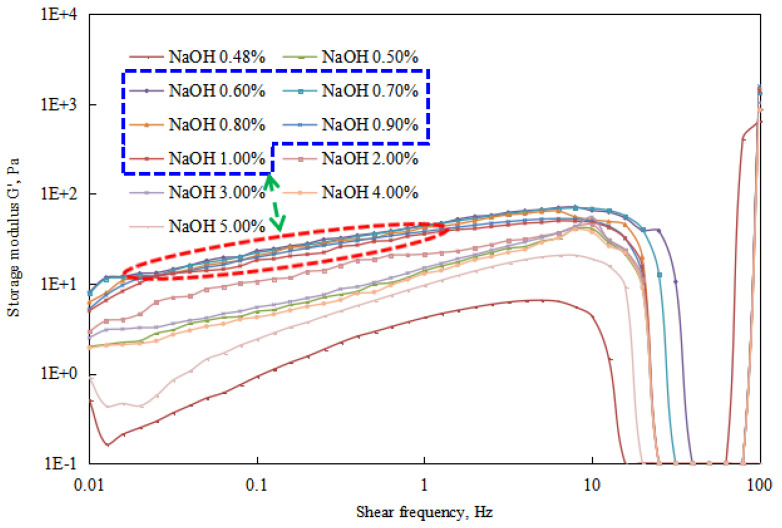
The effect of the NaOH solution on the storage modulus of the W/O emulsion. (The concentration range of NaOH solution marked by the blue line corresponds to the curve marked by the red line).

**Figure 23:**
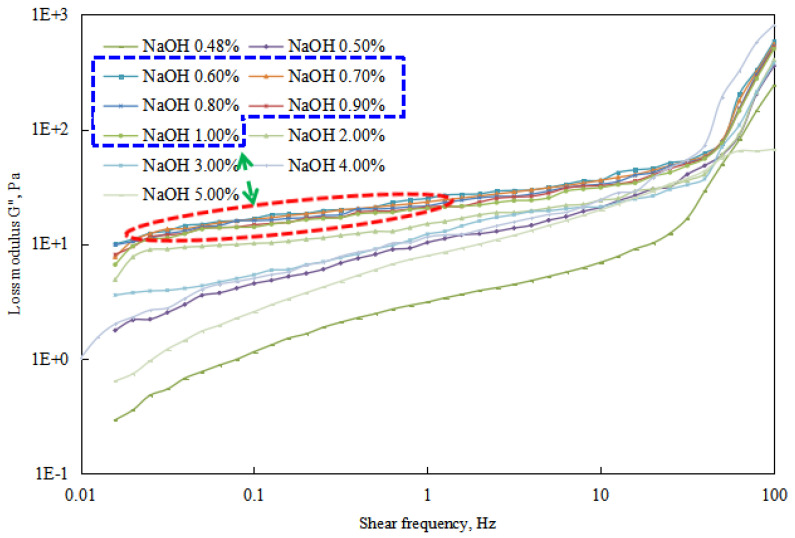
The effect of the NaOH solution on the loss modulus of the W/O emulsion. (The concentration range of NaOH solution marked by the blue line corresponds to the curve marked by the red line).

The changes in energy storage modulus and loss modulus of the reversible emulsion were analyzed through a rheological test of each group of emulsions in the process of alkali conversion (Figure 21, Figure 22 and Figure 23).

By conducting rheological tests on the emulsions formed at different stages of alkali phase inversion in reversible emulsions, experimental results (Figure 21, Figure 22 and Figure 23) indicate as follows. When the amount of NaOH solution is <0.43 vol.%, the reversible emulsion is an O/W emulsion. Due to the absence of structure between the oil droplets in the O/W emulsion, both the storage modulus and loss modulus of the emulsion in this range are relatively low (Figure 21). When the NaOH solution exceeds 0.48 vol.%, the reversible emulsion undergoes phase inversion and becomes a W/O emulsion. As there is structure between the water droplets in the W/O emulsion, the emulsion system exhibits higher viscoelasticity (Figure 22 and Figure 23). At higher stirring speeds, the viscoelasticity of the emulsion shows significant changes, particularly for the O/W emulsion, which experiences a rapid increase. This is due to the rapid shear that disrupts the original structure of the emulsion. The O/W emulsion, lacking structure between the droplets, has poorer stability, and its droplet liquid film structure is more prone to destruction, leading to the formation of a bicontinuous structure. Therefore, higher viscoelasticity is observed in the test results. However, since the structure of the emulsion being studied has been disrupted at this point, it is not further discussed.

Regarding the viscoelasticity test results (Figure 21, Figure 22 and Figure 23) of the W/O emulsion stage during the alkali phase inversion process of the reversible emulsion, the storage modulus and loss modulus of the W/O emulsion in this stage can be arranged from largest to smallest as follows: 0.6% > 0.7% > 0.8% > 0.9% > 1.0% > 2.0% > 3.0% > 0.5% > 4.0% > 5.0% > 0.48%.

The above ordering is consistent with the trend of varying compactness of the structure between water droplets in the W/O emulsion under different NaOH solution conditions during the alkali phase inversion process of the reversible emulsion (Figure 18). In other words, the compactness of the structure between water droplets in the W/O emulsion determines the magnitude of the storage modulus and loss modulus of the emulsion.

Each oil-water system has its most suitable HLB value; that is, the HLB value that can be emulsified into a good W/O emulsion is called HLB-O. Conversely, the HLB value that can be emulsified into an O/W emulsion in good condition is called HLB-W. The more the HLB value of the stable emulsion system deviates from the optimal value, the worse the emulsion state. The focus of reversible emulsion is the emulsifier and the deep reason for the change of properties in the process of reversible emulsion alkali addition is the change of the emulsifier system. Due to the response of the DMOB molecule of the emulsifier to acid/alkali, the proportion of ionic surfactant at the oil-water interface will gradually decrease during the alkali addition process (this can be seen by the gradual decrease of the droplet chargeability (Zeta) with the addition of the alkali solution in the oil-in-water emulsion stage, as shown in Figure 6), which will affect the hydrophilic and lipophilic properties of the oil-water interface emulsifier, and the HLB value of the emulsifier system will gradually decrease with the addition of the alkali. The HLB value of the emulsifier system was higher in the first stage (the O/W emulsion stage), and the hydrophilic and lipophilic characteristics were suitable for stabilizing the O/W emulsion. With the addition of alkali, the HLB value of the emulsifier system gradually decreased, first approaching the optimal HLB value (HLB-W) and then moving away from the optimal HLB value (HLB-W), and the overall state of the emulsion (droplet morphology and stability) also showed a trend of first becoming better and then worsening. The emulsifier system is quite different from HLB-W/HLB-O in the second and third stages, so the emulsion with better performance cannot be stabilized at this stage. With the increase in alkali dosage, the HLB value of the emulsion system further decreased, and in the fourth stage (the W/O emulsion stage), the HLB value of the emulsion system was lower, and the hydrophilic and lipophilic characteristics were suitable for stabilizing the W/O emulsion. With the addition of alkali, the HLB value of the emulsifier system gradually decreased, first approaching the optimal HLB value (HLB-O) and then deviating from the optimal HLB value (HLB-O), and the overall state of the emulsion (droplet morphology and stability) also showed a trend of first becoming better and then worsening.

## 3. Materials and Methods

### 3.1. Materials

**Figure 24:**
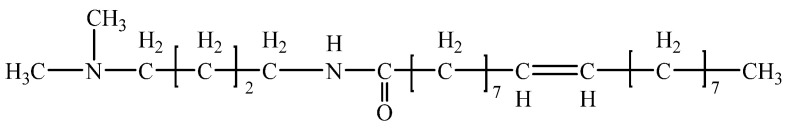
The molecular structure of the DMOB.

Hydrochloric acid, NaOH, analytical grade, provided by China National Pharmaceutical Group Chemical Reagent Co., Ltd., Shanghai, China; Skarlan No. 5 white oil, industrial grade, supplied by Skarlan Petroleum (Chongqing) Co., Ltd., Chongqing, China; DMOB emulsifier (Figure 24), homemade in the laboratory; deionized water.

### 3.2. Experimental Apparatus

DWY-2A Intelligent Electric Stability Tester: Qingdao Xinling Electromechanical Technology Co., Ltd., Qingdao, China; NGJ-2 Mud High-Speed Mixer: Qingdao Jiaonan Analytical Instrument Factory, Qingdao, China; Conductivity Meter DDS-307: Shanghai Jingke; FA1004 Electronic Balance: Shanghai Fangrui Instrument Co., Ltd., Shanghai, China; XSP-11CE Transmitted-Reflected Biological Microscope: Shanghai Changfang Optical Instrument Co., Ltd., Shanghai, China; pH Meter PHSJ-3F: Shanghai Jingke; BROOKFIELD Viscometer: Brookfield Engineering Laboratories, Inc., Shanghai, China; HARK MARS 3 High-Temperature High-Pressure Rheometer: HARK GmbH (Mecklenburg-Vorpommern, Germany); Bettersize2000 Laser Particle Size Analyzer: Dandong Bettersize Instruments Co., Ltd., Dandong, China; JEM-1400Plus Transmission Electron Microscope: JEOL Ltd. (Tokyo, Japan).

### 3.3. Experimental Method

#### 3.3.1. Preparation of Emulsions

Mix 2.5 g DMOB emulsifier and 100 mL Skylan No. 5 white oil. Then, add 100 mL of deionized water. Stir at a speed of 12,000 r/min for 10 min to form the initial W/O emulsion. This is labeled as a Type I emulsion. 

Take 100 mL of Type I emulsion for each group and add different concentrations (0.60 vol.%) of hydrochloric acid with a concentration of 5.00 wt.% (5.00 vol.%). Stir at 12,000 r/min for 5 min. The prepared W/O emulsion is labeled as a type II emulsion.

Due to the rapid phase inversion process of reversible emulsions, it is difficult to study the phase inversion process by controlling stirring times. After the addition of acid/alkali to the emulsion, except for the point of acid/alkali addition, the rest of the emulsion system experiences a rapid increase in acid/alkali concentration with increasing stirring time. Therefore, the change in the reversible emulsion with the increase in the amount of acid/alkali was used to characterize the phase transition process of the reversible emulsion.

Parallel experiments were conducted for different groups. In each group, 100 mL of Type II emulsion was taken, and different volumes of NaOH solution with a concentration of 5 wt.% (ranging from 0 to 5 vol.%) were added to the emulsions. The mixtures were stirred at 12,000 rpm for a certain time. The emulsion breaking voltage, conductivity, static stability, pH value, dispersed droplet morphology, viscosity, rheological properties, and particle size distribution of each group of emulsions were also tested.

The NaOH solution mentioned in this study refers to a 5% (mass fraction) NaOH aqueous solution prepared by mixing NaOH with deionized water.

#### 3.3.2. Preparation of Initial Reversible Emulsion

Let the emulsion stand, observe the separation of oil and water, and record the *volume* of the oil phase and water phase separated from the emulsion over time. The time when an obvious water phase or oil phase precipitates is called the emulsion static stabilization time. Calculate the water and oil separation rate according to the water and oil volumes of the emulsion [29,30]. The separation rates mentioned in this paper are the water and oil evolution rates of the emulsion after standing for 24 h.

(1) $$\mathrm{Water}\;\mathrm{separation}\;\mathrm{rate}=\frac{Volume\;of\;separated\;water\;phase}{Total\;volume\;of\;emulsion}$$

(2) $$Oilseparationrate=\frac{Volume\;of\;separated\;oil\;phase}{Total\;volume\;of\;emulsion}$$

## 4. Conclusions

**Figure 25:**
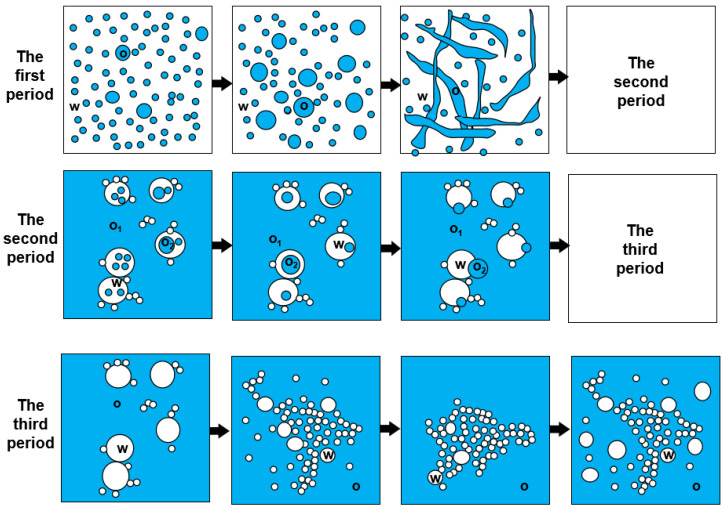
The diagram shows three stages during the alkali-induced phase inversion.

Alkaline on the experimental results, the phase conversion process of a reversible emulsion with alkali is determined. It is O/W emulsion ⟶ bicontinuous structure ⟶ O/W/O emulsion ⟶ W/O emulsion. In the first stage (the amount of NaOH solution ≤ 0.43 vol.%, stirring time 5 min), the overall droplet size of the emulsion shows a decreasing trend, followed by an increasing trend with increasing NaOH solution amounts. In the second stage (the amount of NaOH solution is 0.45 vol.%; stirring time is 5 min), some droplets of the O/W emulsion are disrupted, resulting in the coexistence of the O/W emulsion and a bicontinuous structure within the emulsion. In the third stage (NaOH solution amount of 0.48 vol.%), an O/W/O emulsion is formed, and with increasing stirring time, the internal oil droplets gradually separate from the water droplets, eventually forming a W/O emulsion. In the fourth stage (0.50 vol.% ≤ NaOH solution amount ≤ 5.00 vol.%, stirring time 5 min), a W/O emulsion is formed. The compactness of the structure between water droplets in the emulsion initially increases and then decreases with increasing NaOH solution amount. Throughout this process, the overall droplet size of the emulsion shows a decreasing trend, followed by an increasing trend (Figure 25).

## Figures and Tables

**Table 1:** The experimental methods and functions used in this article.

Experimental Method	Function
microscope, Cryo-TEM, laser particle sizer	The change of the micro-properties
Zeta potentiometer	The change in the distribution of the reversible inverted emulsifier at the oil-water interface
Breaking voltage tester, Conductivity meter, pH meter, Viscometer, Rheometer	The change of the macro-properties

## Data Availability

The data presented in this study are available wholly within the manuscript.
